# Crystal structure of 2-(di­phenyl­phos­phanyl)phenyl 4-(hy­droxy­meth­yl)benzoate

**DOI:** 10.1107/S1600536814024623

**Published:** 2014-11-26

**Authors:** Constantin Mamat, Anke Flemming, Martin Köckerling

**Affiliations:** a Institut für Radiopharmazeutische Krebsforschung, Bautzner Landstr. 400, D-01328 Dresden, Germany; b Universität Rostock, Institut für Chemie, Anorganische Festkörperchemie, Albert-Einstein-Str. 3a, D-18059 Rostock, Germany

**Keywords:** crystal structure, benzoate functionalized 2-(di­phenyl­phosphano)phenol derivative, hydrogen bonding

## Abstract

The title compound, C_26_H_21_O_3_P, was obtained as by-product due to the hydrolysis of the desired tosyl­ated compound. The dihedral angles between the three aromatic rings attached to the P atom lie in the range 78.1 (1)–87.6 (1)°. The hy­droxy­methyl group is disordered between two conformations in a 0.719 (9):0.281 (9) ratio. The hy­droxy H atom is not involved in inter­molecular inter­actions, while the hy­droxy O atom serves as a donor for weak C—H⋯O hydrogen bonds, which link the mol­ecules into chains propagating in [0-11].

## Related literature   

For a general introduction to the chemistry and radiochemistry of benzoate functionalized 2-(di­phenyl­phosphanyl)phenol derivatives, see: Mamat *et al.* (2009 ▶); Pretze *et al.* (2010 ▶). For applications of chloro­methyl and hy­droxy­methyl benzoates, see: Mamat *et al.* (2011 ▶); Wodtke *et al.* (2015 ▶).
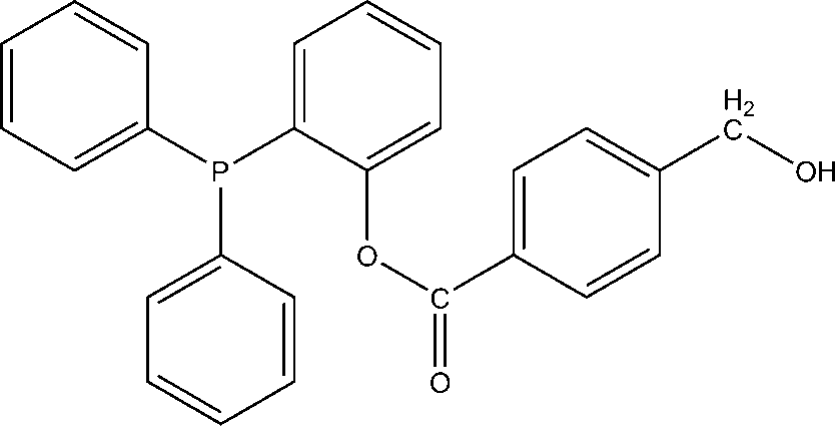


## Experimental   

### Crystal data  

C_26_H_21_O_3_P
*M* *_r_* = 412.40
Triclinic, 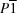

*a* = 9.8197 (3) Å
*b* = 10.6503 (3) Å
*c* = 11.6744 (3) Å
α = 74.245 (1)°
β = 80.428 (1)°
γ = 66.493 (1)°
*V* = 1075.23 (5) Å^3^
*Z* = 2
Mo *K*α radiation
μ = 0.15 mm^−1^
*T* = 296 K
0.41 × 0.39 × 0.31 mm

### Data collection  

Bruker APEXII CCD diffractometer
Absorption correction: multi-scan (*SADABS*; Bruker, 2007 ▶) *T*_min_ = 0.940, *T*_max_ = 0.954
40695 measured reflections
12072 independent reflections
7599 reflections with *I* > 2σ(*I*)
*R*_int_ = 0.019

### Refinement  

*R*[*F* ^2^ > 2σ(*F* ^2^)] = 0.051
*wR*(*F* ^2^) = 0.176
*S* = 1.02
12072 reflections
292 parameters
H-atom parameters constrained
Δρ_max_ = 0.43 e Å^−3^
Δρ_min_ = −0.20 e Å^−3^

### 

Data collection: *APEX2* (Bruker, 2007 ▶); cell refinement: *SAINT* (Bruker, 2007 ▶); data reduction: *SAINT*; program(s) used to solve structure: *SHELXS97* (Sheldrick, 2008 ▶); program(s) used to refine structure: *SHELXL2014*/1 (Sheldrick, 2008 ▶); molecular graphics: *SHELXTL* (Sheldrick, 2008 ▶); software used to prepare material for publication: *SHELXTL*.

## Supplementary Material

Crystal structure: contains datablock(s) I. DOI: 10.1107/S1600536814024623/cv5473sup1.cif

Structure factors: contains datablock(s) I. DOI: 10.1107/S1600536814024623/cv5473Isup2.hkl

**Fig. 1.**
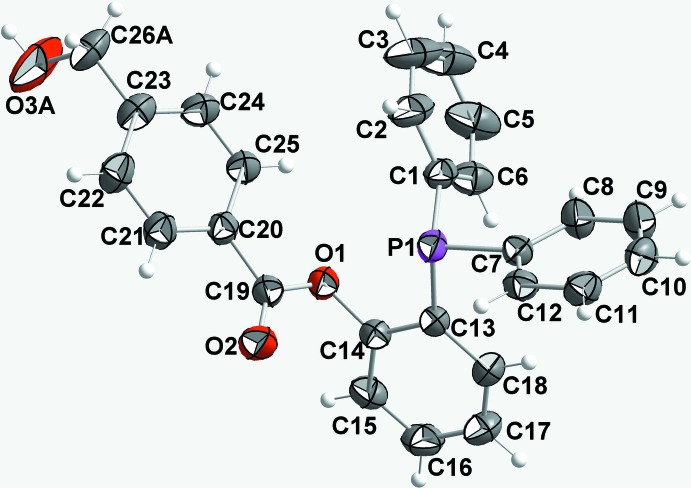
The molecular structure of the title compound, with the atom labels and 50% probability displacement ellipsoids. Only the major component of the disordered hydroxymethyl group is shown.

**Fig. 2.**
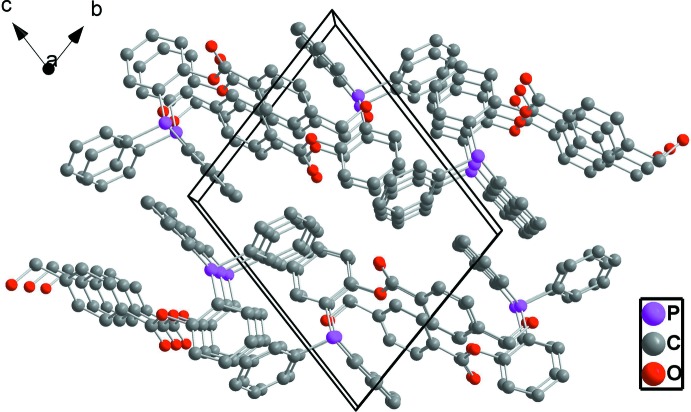
A portion of the crystal packing viewed approximately down the a axis. H atoms have been omitted for clarity.

. DOI: 10.1107/S1600536814024623/cv5473fig1.tif
The mol­ecular structure of the title compound, with the atom labels and 50% probability displacement ellipsoids. Only the major component of the disordered hy­droxy­methyl group is shown.

a . DOI: 10.1107/S1600536814024623/cv5473fig2.tif
A portion of the crystal packing viewed approximately down the *a* axis. H atoms have been omitted for clarity.

CCDC reference: 1033495

Additional supporting information:  crystallographic information; 3D view; checkCIF report

## Figures and Tables

**Table 1:** Hydrogen-bond geometry (, )

*D*H*A*	*D*H	H*A*	*D* *A*	*D*H*A*
C18H18*A*O3*A* ^i^	0.93	2.52	3.143(5)	125
C18H18*A*O3*B* ^i^	0.93	2.62	3.408(9)	143

Symmetry code: (i) 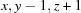.

## supplementary crystallographic information

### S1. Comment

Benzoate functionalized 2-(diphenylphosphano)phenol derivatives are important starting materials for various labeling purposes using the traceless Staudinger Ligation (Mamat *et al.*, 2009). 4-(Halomethyl) and 4-(hydroxymethyl)benzoates were developed, especially, for the insertion of fluorescence markers (Wodtke *et al.*, 2015) or for the introduction of radiolabels such as fluorine-18 (Mamat *et al.*, 2011). For the later purpose, good leaving groups were required. The title compound was obtained as by-product after the tosylation step during the preparation of the tosylated title compound which was further used as precursor for radiofluorinations.

### S2. Experimental

2-(Diphenylphosphano)phenyl-4-(iodomethyl)benzoate was dissolved in acetonitrile and treated with AgOTs in the dark. After 16 h, the precipitate was filtered and the residue was purified by column chromatography after removal of the solvent. The title compound was obtained as by-product due to the hydrolysis of the desired tosylated compound during the purification step.

Colorless crystals of the title compound were obtained by crystallization from ethyl acetate/petroleum ether after chromatographic separation.

### S3. Refinement

All hydrogen atoms were placed on geometrically calculated positions [C—H 0.93–0.97 Å; O—H 0.82 Å], and refined using a riding model, with U_iso_(H) = 1.2 – 1.5 U_eq_ of the parent atom.

The hydroxymethyl group disordered between two orientations was treated using a split model with the occupancies refined to 0.719 (9)/0.281 (9).

### Crystal data

**Table:**

C_26_H_21_O_3_P	*F*(000) = 432
*M**_r_* = 412.40	*D*_x_ = 1.274 Mg m^−^^3^
Triclinic, *P*1	Melting point: 437 K
*a* = 9.8197 (3) Å	Mo *K*α radiation, λ = 0.71073 Å
*b* = 10.6503 (3) Å	Cell parameters from 9899 reflections
*c* = 11.6744 (3) Å	θ = 2.7–35.0°
α = 74.245 (1)°	µ = 0.15 mm^−^^1^
β = 80.428 (1)°	*T* = 296 K
γ = 66.493 (1)°	Block, colourless
*V* = 1075.23 (5) Å^3^	0.41 × 0.39 × 0.31 mm
*Z* = 2	

### Data collection

**Table:**

Bruker APEXII CCD diffractometer	12072 independent reflections
Radiation source: fine-focus sealed tube	7599 reflections with *I* > 2σ(*I*)
Graphite monochromator	*R*_int_ = 0.019
φ and ω scans	θ_max_ = 38.6°, θ_min_ = 2.3°
Absorption correction: multi-scan (*SADABS*; Bruker, 2007)	*h* = −17→17
*T*_min_ = 0.940, *T*_max_ = 0.954	*k* = −18→18
40695 measured reflections	*l* = −20→20

### Refinement

**Table:**

Refinement on *F*^2^	Primary atom site location: structure-invariant direct methods
Least-squares matrix: full	Secondary atom site location: difference Fourier map
*R*[*F*^2^ > 2σ(*F*^2^)] = 0.051	Hydrogen site location: inferred from neighbouring sites
*wR*(*F*^2^) = 0.176	H-atom parameters constrained
*S* = 1.02	*w* = 1/[σ^2^(*F*_o_^2^) + (0.0924*P*)^2^ + 0.0784*P*] where *P* = (*F*_o_^2^ + 2*F*_c_^2^)/3
12072 reflections	(Δ/σ)_max_ = 0.001
292 parameters	Δρ_max_ = 0.43 e Å^−^^3^
0 restraints	Δρ_min_ = −0.20 e Å^−^^3^

### Special details

**Table:**

Geometry. All e.s.d.'s (except the e.s.d. in the dihedral angle between two l.s. planes) are estimated using the full covariance matrix. The cell e.s.d.'s are taken into account individually in the estimation of e.s.d.'s in distances, angles and torsion angles; correlations between e.s.d.'s in cell parameters are only used when they are defined by crystal symmetry. An approximate (isotropic) treatment of cell e.s.d.'s is used for estimating e.s.d.'s involving l.s. planes.

### Fractional atomic coordinates and isotropic or equivalent isotropic displacement parameters (Å^2^)

**Table:**

	*x*	*y*	*z*	*U*_iso_*/*U*_eq_	Occ. (<1)
P1	0.84403 (3)	0.11854 (2)	0.24387 (2)	0.04106 (7)	
C1	0.75048 (12)	0.12573 (10)	0.11847 (8)	0.0442 (2)	
C2	0.82426 (17)	0.14309 (16)	0.00583 (11)	0.0667 (3)	
H2A	0.9184	0.1469	−0.0018	0.080*	
C3	0.7585 (2)	0.1547 (2)	−0.09483 (13)	0.0935 (6)	
H3A	0.8084	0.1663	−0.1696	0.112*	
C4	0.6202 (2)	0.1492 (2)	−0.08396 (16)	0.0973 (7)	
H4A	0.5766	0.1564	−0.1515	0.117*	
C5	0.54466 (18)	0.13304 (19)	0.02639 (15)	0.0779 (5)	
H5A	0.4503	0.1299	0.0328	0.094*	
C6	0.60923 (13)	0.12146 (14)	0.12780 (11)	0.0553 (3)	
H6A	0.5580	0.1108	0.2021	0.066*	
C7	0.92219 (10)	−0.06956 (9)	0.31351 (8)	0.03940 (17)	
C8	0.87987 (13)	−0.17150 (11)	0.29290 (11)	0.0512 (2)	
H8A	0.8029	−0.1446	0.2438	0.061*	
C9	0.95166 (15)	−0.31296 (12)	0.34509 (12)	0.0571 (3)	
H9A	0.9223	−0.3801	0.3311	0.069*	
C10	1.06669 (14)	−0.35384 (12)	0.41781 (11)	0.0541 (2)	
H10A	1.1150	−0.4485	0.4521	0.065*	
C11	1.10998 (14)	−0.25441 (12)	0.43962 (10)	0.0525 (2)	
H11A	1.1863	−0.2820	0.4896	0.063*	
C12	1.03918 (12)	−0.11301 (11)	0.38674 (9)	0.0459 (2)	
H12A	1.0701	−0.0466	0.4003	0.055*	
C13	0.68656 (10)	0.18502 (9)	0.34901 (8)	0.03825 (16)	
C14	0.60240 (11)	0.32942 (10)	0.32357 (8)	0.03978 (17)	
C15	0.48966 (13)	0.39328 (12)	0.40090 (11)	0.0517 (2)	
H15A	0.4360	0.4897	0.3813	0.062*	
C16	0.45770 (15)	0.31154 (15)	0.50816 (11)	0.0601 (3)	
H16A	0.3827	0.3531	0.5616	0.072*	
C17	0.53712 (15)	0.16834 (15)	0.53568 (11)	0.0597 (3)	
H17A	0.5144	0.1137	0.6073	0.072*	
C18	0.65102 (13)	0.10505 (11)	0.45706 (9)	0.0482 (2)	
H18A	0.7039	0.0085	0.4768	0.058*	
O1	0.62903 (8)	0.40814 (7)	0.21064 (6)	0.04425 (15)	
C19	0.71938 (11)	0.47982 (10)	0.20030 (9)	0.04134 (18)	
O2	0.76590 (12)	0.48959 (11)	0.28545 (8)	0.0627 (2)	
C20	0.75123 (11)	0.54141 (9)	0.07365 (8)	0.04067 (17)	
C21	0.82940 (13)	0.63110 (12)	0.04775 (11)	0.0508 (2)	
H21A	0.8608	0.6509	0.1093	0.061*	
C22	0.86029 (14)	0.69063 (14)	−0.06974 (12)	0.0574 (3)	
H22A	0.9117	0.7511	−0.0867	0.069*	
C23	0.81488 (13)	0.66056 (13)	−0.16261 (10)	0.0543 (2)	
C24	0.73914 (15)	0.56961 (14)	−0.13621 (10)	0.0553 (3)	
H24A	0.7097	0.5483	−0.1980	0.066*	
C25	0.70666 (13)	0.51005 (11)	−0.01914 (9)	0.0477 (2)	
H25A	0.6554	0.4495	−0.0025	0.057*	
C26A	0.8460 (5)	0.7261 (4)	−0.2899 (3)	0.0678 (9)	0.719 (9)
H26A	0.9479	0.7215	−0.3026	0.081*	0.719 (9)
H26B	0.8320	0.6765	−0.3427	0.081*	0.719 (9)
O3A	0.7490 (5)	0.8636 (4)	−0.3130 (3)	0.126 (2)	0.719 (9)
H3B	0.7261	0.8919	−0.2511	0.189*	0.719 (9)
C26B	0.840 (2)	0.751 (3)	−0.2877 (14)	0.141 (8)	0.281 (9)
H26C	0.8470	0.8364	−0.2786	0.170*	0.281 (9)
H26D	0.9335	0.6990	−0.3263	0.170*	0.281 (9)
O3B	0.7332 (11)	0.7830 (17)	−0.3523 (7)	0.132 (5)	0.281 (9)
H3C	0.7558	0.7259	−0.3940	0.198*	0.281 (9)

### Atomic displacement parameters (Å^2^)

**Table:**

	*U*^11^	*U*^22^	*U*^33^	*U*^12^	*U*^13^	*U*^23^
P1	0.04208 (13)	0.03810 (12)	0.04163 (12)	−0.01697 (9)	0.00118 (9)	−0.00612 (9)
C1	0.0481 (5)	0.0389 (4)	0.0357 (4)	−0.0074 (3)	0.0001 (3)	−0.0079 (3)
C2	0.0682 (8)	0.0736 (8)	0.0416 (5)	−0.0141 (6)	0.0117 (5)	−0.0146 (5)
C3	0.1029 (13)	0.1108 (14)	0.0398 (6)	−0.0092 (11)	0.0030 (7)	−0.0262 (8)
C4	0.0991 (13)	0.1105 (14)	0.0591 (8)	0.0073 (11)	−0.0291 (8)	−0.0398 (9)
C5	0.0613 (7)	0.0883 (10)	0.0766 (9)	0.0000 (7)	−0.0249 (7)	−0.0375 (8)
C6	0.0486 (5)	0.0615 (6)	0.0498 (5)	−0.0094 (5)	−0.0064 (4)	−0.0187 (5)
C7	0.0392 (4)	0.0384 (4)	0.0395 (4)	−0.0136 (3)	−0.0019 (3)	−0.0088 (3)
C8	0.0527 (5)	0.0437 (5)	0.0597 (6)	−0.0194 (4)	−0.0186 (5)	−0.0047 (4)
C9	0.0675 (7)	0.0415 (5)	0.0664 (7)	−0.0238 (5)	−0.0177 (6)	−0.0052 (4)
C10	0.0591 (6)	0.0421 (5)	0.0527 (6)	−0.0129 (4)	−0.0110 (5)	−0.0021 (4)
C11	0.0551 (6)	0.0546 (6)	0.0439 (5)	−0.0159 (5)	−0.0145 (4)	−0.0051 (4)
C12	0.0523 (5)	0.0484 (5)	0.0404 (4)	−0.0188 (4)	−0.0078 (4)	−0.0125 (4)
C13	0.0449 (4)	0.0381 (4)	0.0344 (3)	−0.0188 (3)	−0.0009 (3)	−0.0081 (3)
C14	0.0463 (4)	0.0383 (4)	0.0375 (4)	−0.0177 (3)	−0.0045 (3)	−0.0089 (3)
C15	0.0520 (5)	0.0481 (5)	0.0541 (6)	−0.0131 (4)	−0.0014 (4)	−0.0199 (4)
C16	0.0589 (6)	0.0716 (8)	0.0518 (6)	−0.0237 (6)	0.0120 (5)	−0.0274 (6)
C17	0.0692 (7)	0.0698 (7)	0.0421 (5)	−0.0342 (6)	0.0117 (5)	−0.0121 (5)
C18	0.0588 (6)	0.0457 (5)	0.0397 (4)	−0.0242 (4)	0.0025 (4)	−0.0050 (4)
O1	0.0553 (4)	0.0405 (3)	0.0401 (3)	−0.0223 (3)	−0.0096 (3)	−0.0036 (2)
C19	0.0464 (4)	0.0369 (4)	0.0414 (4)	−0.0154 (3)	−0.0063 (3)	−0.0081 (3)
O2	0.0853 (6)	0.0788 (6)	0.0444 (4)	−0.0514 (5)	−0.0092 (4)	−0.0111 (4)
C20	0.0429 (4)	0.0364 (4)	0.0407 (4)	−0.0136 (3)	−0.0069 (3)	−0.0050 (3)
C21	0.0548 (6)	0.0529 (5)	0.0501 (5)	−0.0273 (5)	−0.0088 (4)	−0.0057 (4)
C22	0.0560 (6)	0.0610 (7)	0.0574 (6)	−0.0317 (5)	−0.0044 (5)	−0.0010 (5)
C23	0.0505 (5)	0.0569 (6)	0.0452 (5)	−0.0173 (5)	−0.0019 (4)	−0.0005 (4)
C24	0.0649 (7)	0.0610 (6)	0.0405 (5)	−0.0248 (5)	−0.0080 (4)	−0.0074 (4)
C25	0.0570 (6)	0.0466 (5)	0.0428 (5)	−0.0229 (4)	−0.0076 (4)	−0.0072 (4)
C26A	0.075 (2)	0.0728 (15)	0.0438 (12)	−0.0333 (13)	0.0023 (11)	0.0108 (10)
O3A	0.141 (3)	0.0907 (19)	0.0719 (14)	−0.0052 (16)	−0.0068 (16)	0.0415 (13)
C26B	0.086 (9)	0.26 (2)	0.086 (8)	−0.061 (11)	−0.008 (6)	−0.061 (10)
O3B	0.136 (5)	0.201 (11)	0.070 (4)	−0.120 (6)	−0.056 (4)	0.069 (5)

### Geometric parameters (Å, º)

**Table:**

P1—C1	1.823 (1)	C15—H15A	0.9300
P1—C7	1.8330 (9)	C16—C17	1.3805 (19)
P1—C13	1.8347 (9)	C16—H16A	0.9300
C1—C6	1.391 (2)	C17—C18	1.3931 (16)
C1—C2	1.3950 (15)	C17—H17A	0.9300
C2—C3	1.387 (2)	C18—H18A	0.9300
C2—H2A	0.9300	O1—C19	1.356 (1)
C3—C4	1.366 (3)	C19—O2	1.205 (1)
C3—H3A	0.9300	C19—C20	1.4824 (13)
C4—C5	1.380 (3)	C20—C21	1.3943 (14)
C4—H4A	0.9300	C20—C25	1.3955 (14)
C5—C6	1.3881 (18)	C21—C22	1.3850 (17)
C5—H5A	0.9300	C21—H21A	0.9300
C6—H6A	0.9300	C22—C23	1.3930 (18)
C7—C8	1.3944 (14)	C22—H22A	0.9300
C7—C12	1.3973 (13)	C23—C24	1.3866 (18)
C8—C9	1.3911 (15)	C23—C26A	1.498 (3)
C8—H8A	0.9300	C23—C26B	1.560 (18)
C9—C10	1.3828 (17)	C24—C25	1.3847 (15)
C9—H9A	0.9300	C24—H24A	0.9300
C10—C11	1.3815 (17)	C25—H25A	0.9300
C10—H10A	0.9300	C26A—O3A	1.372 (5)
C11—C12	1.3911 (15)	C26A—H26A	0.9700
C11—H11A	0.9300	C26A—H26B	0.9700
C12—H12A	0.9300	O3A—H3B	0.8200
C13—C18	1.3949 (13)	C26B—O3B	1.28 (2)
C13—C14	1.3986 (13)	C26B—H26C	0.9700
C14—C15	1.3796 (15)	C26B—H26D	0.9700
C14—O1	1.403 (1)	O3B—H3C	0.8200
C15—C16	1.3856 (19)		
			
C1—P1—C7	103.65 (4)	C17—C16—C15	120.01 (10)
C1—P1—C13	102.01 (4)	C17—C16—H16A	120.0
C7—P1—C13	102.34 (4)	C15—C16—H16A	120.0
C6—C1—C2	118.77 (11)	C16—C17—C18	120.54 (11)
C6—C1—P1	124.24 (8)	C16—C17—H17A	119.7
C2—C1—P1	116.93 (10)	C18—C17—H17A	119.7
C3—C2—C1	120.59 (16)	C17—C18—C13	120.66 (10)
C3—C2—H2A	119.7	C17—C18—H18A	119.7
C1—C2—H2A	119.7	C13—C18—H18A	119.7
C4—C3—C2	119.88 (15)	C19—O1—C14	118.46 (7)
C4—C3—H3A	120.1	O2—C19—O1	122.61 (9)
C2—C3—H3A	120.1	O2—C19—C20	126.05 (9)
C3—C4—C5	120.58 (14)	O1—C19—C20	111.34 (8)
C3—C4—H4A	119.7	C21—C20—C25	119.77 (10)
C5—C4—H4A	119.7	C21—C20—C19	118.68 (9)
C4—C5—C6	120.09 (17)	C25—C20—C19	121.54 (9)
C4—C5—H5A	120.0	C22—C21—C20	119.97 (10)
C6—C5—H5A	120.0	C22—C21—H21A	120.0
C5—C6—C1	120.08 (13)	C20—C21—H21A	120.0
C5—C6—H6A	120.0	C21—C22—C23	120.42 (11)
C1—C6—H6A	120.0	C21—C22—H22A	119.8
C8—C7—C12	118.45 (9)	C23—C22—H22A	119.8
C8—C7—P1	124.96 (7)	C24—C23—C22	119.28 (11)
C12—C7—P1	116.47 (7)	C24—C23—C26A	120.0 (2)
C9—C8—C7	120.66 (10)	C22—C23—C26A	120.7 (2)
C9—C8—H8A	119.7	C24—C23—C26B	126.8 (7)
C7—C8—H8A	119.7	C22—C23—C26B	113.4 (8)
C10—C9—C8	120.04 (10)	C25—C24—C23	120.91 (11)
C10—C9—H9A	120.0	C25—C24—H24A	119.5
C8—C9—H9A	120.0	C23—C24—H24A	119.5
C11—C10—C9	120.18 (10)	C24—C25—C20	119.63 (10)
C11—C10—H10A	119.9	C24—C25—H25A	120.2
C9—C10—H10A	119.9	C20—C25—H25A	120.2
C10—C11—C12	119.87 (10)	O3A—C26A—C23	107.7 (3)
C10—C11—H11A	120.1	O3A—C26A—H26A	110.2
C12—C11—H11A	120.1	C23—C26A—H26A	110.2
C11—C12—C7	120.78 (10)	O3A—C26A—H26B	110.2
C11—C12—H12A	119.6	C23—C26A—H26B	110.2
C7—C12—H12A	119.6	H26A—C26A—H26B	108.5
C18—C13—C14	117.14 (9)	C26A—O3A—H3B	109.5
C18—C13—P1	125.19 (8)	O3B—C26B—C23	110.1 (12)
C14—C13—P1	117.47 (7)	O3B—C26B—H26C	109.6
C15—C14—C13	122.70 (9)	C23—C26B—H26C	109.6
C15—C14—O1	119.88 (9)	O3B—C26B—H26D	109.6
C13—C14—O1	117.26 (8)	C23—C26B—H26D	109.6
C14—C15—C16	118.95 (11)	H26C—C26B—H26D	108.2
C14—C15—H15A	120.5	C26B—O3B—H3C	109.5
C16—C15—H15A	120.5		

### Hydrogen-bond geometry (Å, º)

**Table:**

*D*—H···*A*	*D*—H	H···*A*	*D*···*A*	*D*—H···*A*
C18—H18*A*···O3*A*^i^	0.93	2.52	3.143 (5)	125
C18—H18*A*···O3*B*^i^	0.93	2.62	3.408 (9)	143

Symmetry code: (i) *x*, *y*−1, *z*+1.
