# Supplementary figures and images for: Crystal structure of 2-(di­phenyl­phos­phanyl)phenyl 4-(hy­droxy­meth­yl)benzoate
Source: Acta Crystallogr Sect E Struct Rep Online. 2014 Nov 26;70(Pt 12):o1288–9. doi: 10.1107/S1600536814024623 (PMC4257391; doi:10.1107/S1600536814024623)

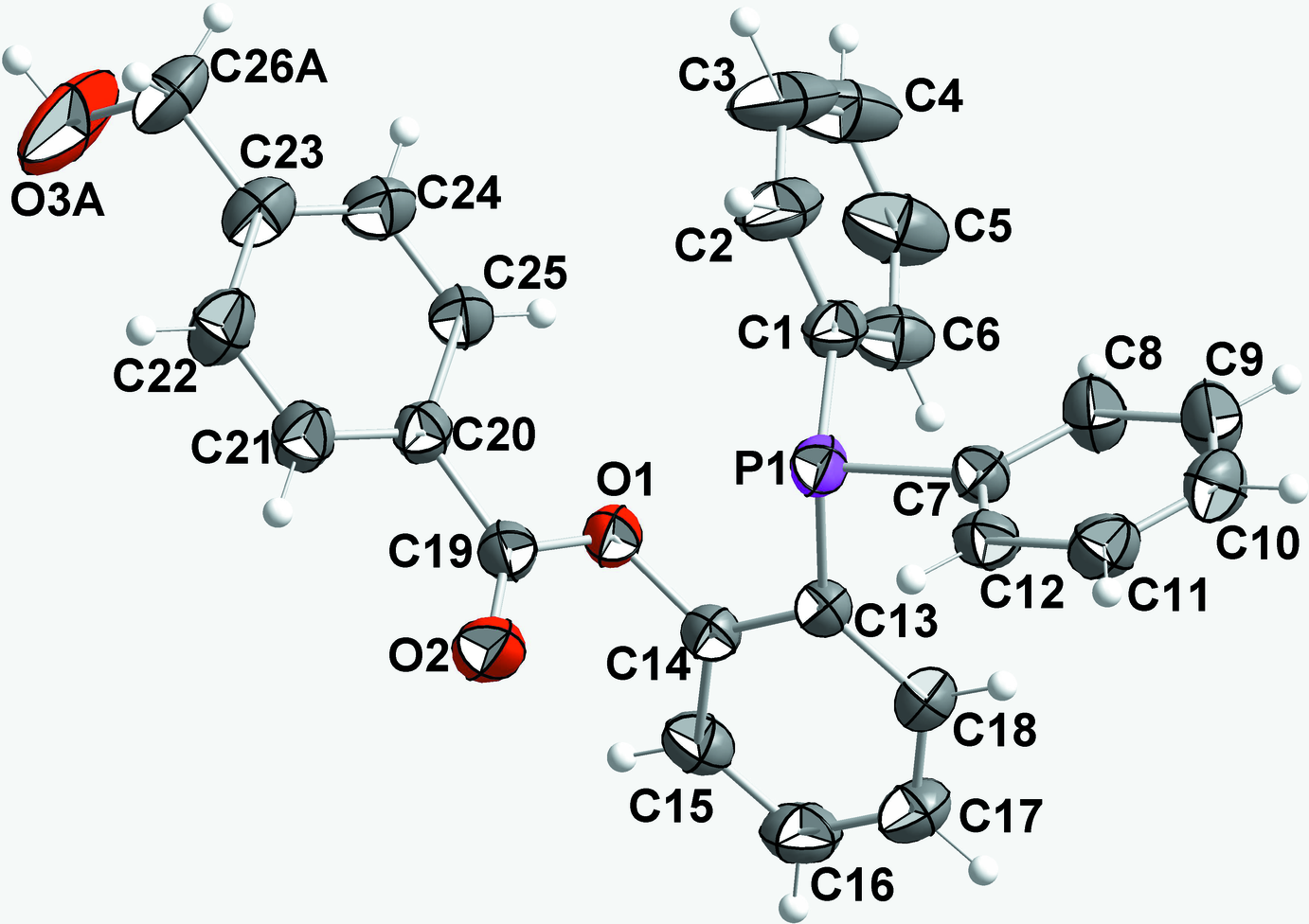

Supplement: Supplementary file 3 [file e-70-o1288-fig1.tif]

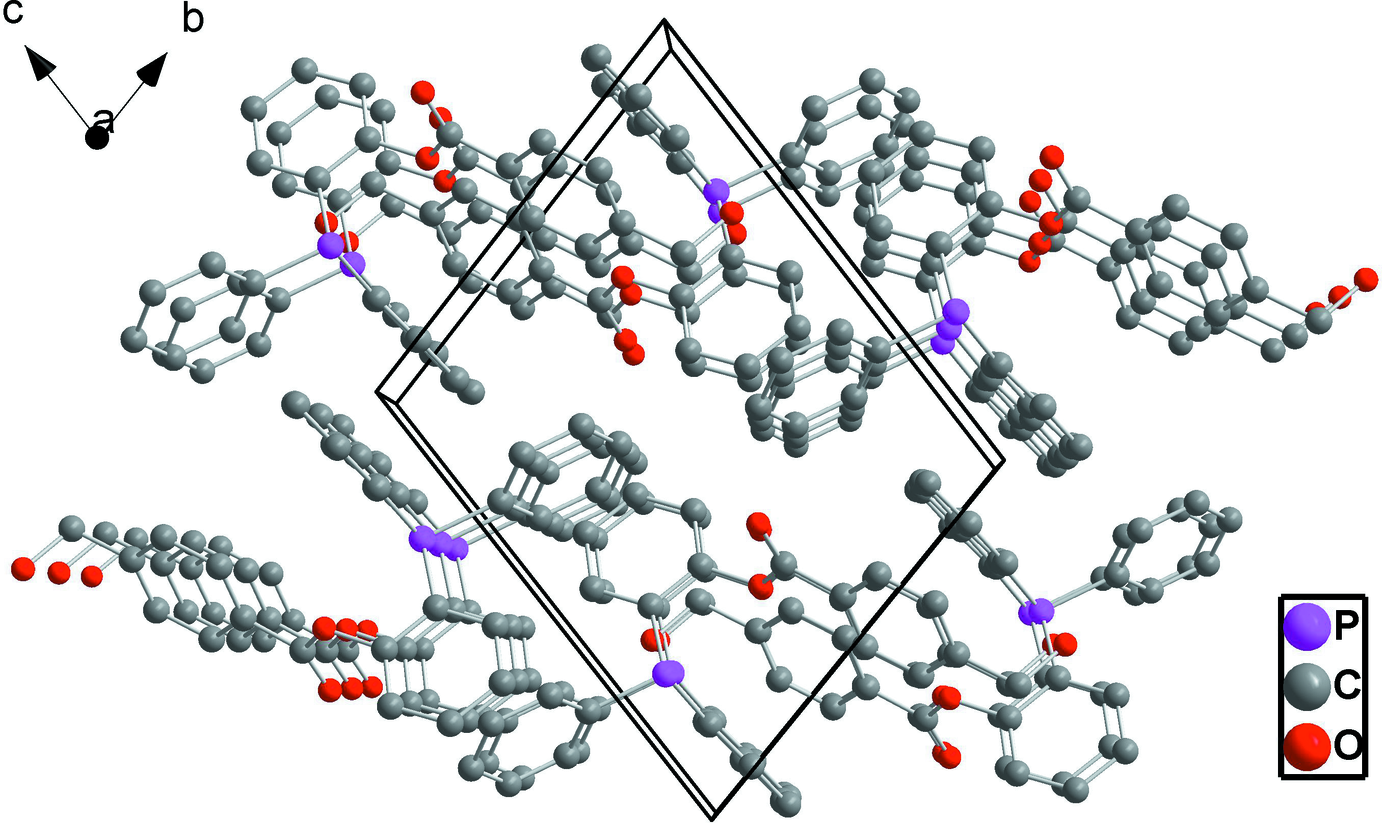

Supplement: Supplementary file 4 [file e-70-o1288-fig2.tif]
